# Nutritional life cycle assessment for healthy and sustainable food systems: evidence and policy insights from Africa and Asia

**DOI:** 10.3389/fnut.2026.1774865

**Published:** 2026-02-18

**Authors:** Graham A. McAuliffe, Flaminia Ortenzi, Jolieke C. van der Pols, Thomas Nemecek, Jessica Colston, Ty Beal

**Affiliations:** 1 Harper Food Innovation, Harper Adams University, Newport, United Kingdom; 2 Knowledge Leadership, Global Alliance for Improved Nutrition (GAIN), Geneva, Switzerland; 3 School of Exercise and Nutrition Sciences and the Centre for Agriculture and the Bioeconomy, Queensland University of Technology (QUT), Brisbane, QLD, Australia; 4 Life Cycle Assessment Research Group, Agroscope, Zurich, Switzerland; 5 Programme Services Team, Global Alliance for Improved Nutrition (GAIN), London, United Kingdom; 6 Knowledge Leadership, Global Alliance for Improved Nutrition (GAIN), Washington, DC, United States

**Keywords:** environmental impact, food policy, low- and middle-income countries, nLCA, nutrient profiling, nutritional life cycle assessment, sustainable food systems

## Abstract

Integrating nutritional value into Life Cycle Assessment (LCA) is essential for developing food system policies and interventions that simultaneously address environmental sustainability and human health. This Perspective explores recent conceptual and empirical evolutions in nutritional LCA (nLCA), drawing on expert talks, interdisciplinary stakeholder deliberations, and case studies presented at the 23rd International Union of Nutritional Sciences – International Congress of Nutrition, held in Paris in August 2025. We discuss methodological frameworks for incorporating nutritional quality into environmental footprint modelling, focusing on the selection of functional units and application of holistic nutrient profiling systems, such as the Nutritional Value Score. Case studies from Africa and Asia demonstrate the utility of nLCA to identify highly nutritious, lower-impact foods that mass- or energy-based denominators often overlook under attributional LCA. We argue that while plant-source foods frequently exhibit lower footprints, certain animal-source foods (such as small fish, dairy, eggs, and organ meats) can also be competitive when evaluated per unit of nutritional value. Finally, we highlight persistent challenges, including regional data gaps, lack of harmonisation in nutritional functional units, scope limitations, and risks of overinterpreting small differences in impact scores. While methodological refinement is still required, we conclude that nLCA offers a promising route for aligning agricultural, health, and environmental objectives, facilitating the development of more coherent food systems policies and programmes.

## 1 Introduction

The urgent need to align environmental stewardship with global health priorities has recently placed food systems transformation at the centre of international policy debates (1–3). Life Cycle Assessment (LCA) often serves as a prominent methodology for quantifying the environmental burdens of goods or services. However, when applied to food systems, conventional mass- or energy-based LCA may yield incomplete, potentially misleading insights for decision-makers because it fails to account for the ultimate purpose of food: delivering adequate nutrition (4). We argue that for LCA to serve as a meaningful tool for food systems policy and interventions, it must be adapted to incorporate nutritional value – a framework increasingly recognised as nutritional LCA (nLCA) (4).

This perspective draws on evidence presented at a dedicated symposium on the evolution of nLCA within food systems held during the 23rd International Union of Nutritional Sciences – International Congress of Nutrition (IUNS-ICN) in Paris (August 2025; https://www.icn2025.org/). The session convened transdisciplinary and cross-sectoral experts to address *both* methodological advances *and* persistent challenges in applying nLCA to food and dietary assessments, with a particular focus on low- and middle-income country (LMIC) settings.

The symposium underscored that the move towards nLCA is not merely a technical adjustment but a necessary conceptual shift that allows for the prioritisation of supply chains, foods, and dietary patterns in ways that balance environmental goals with improved health and nutrition outcomes. The invited speakers covered the theoretical foundations of nLCA (4); the critical role of functional unit selection in determining the results (5–11); the use of holistic nutrient profiling systems such as the Nutritional Value Score (NVS) in environmental impact modelling (12); the application of combined *enviro-nutritional* footprint scores to local policy and programmatic decision-making (13); and current evidence gaps, limitations, and recommendations for future research (4, 14).

While significant methodological issues and data availability constraints remain, empirical case studies from diverse contexts – spanning LMIC (including, for instance, Indonesia, Kenya, and Rwanda) and high-income country (HIC; such as New Zealand, the United Kingdom, and Switzerland) settings – illustrate that nLCA can effectively reveal key trade-offs and synergies between nourishing a growing global population and minimising food-related environmental burdens (8, 13, 15, 16). By shifting the analytical lens from mass or energy to nutritional quality, nLCA offers a powerful framework for optimising food systems toward enhancing both human and planetary health.

## 2 The case for nutritional functional units

Designing healthier and more sustainable food systems requires effectively integrating environmental impacts with nutritional needs (1–3). Thus, the symposium opened with a strong rationale for nLCA as a data-driven tool to reconcile these often-conflicting objectives. Of note, large variability in nutritional and environmental priorities across geographic regions necessitates the selection of appropriate functional units that reflect the intended purpose of an assessment – whether mass- or calorie-based, nutrient-specific (e.g., protein, calcium, vitamin A), or composite nutritional indices (e.g., NVS and, more commonly, the Nutrient Rich Food Index) (4–11). Crucially, the chosen metric must be relevant to the specific study context, taking into account the target population’s nutritional requirements and the range and types of foods being compared. To this end, tailored weighting systems can be applied to composite indices to better reflect local needs (8, 17).

**Figure 1:**
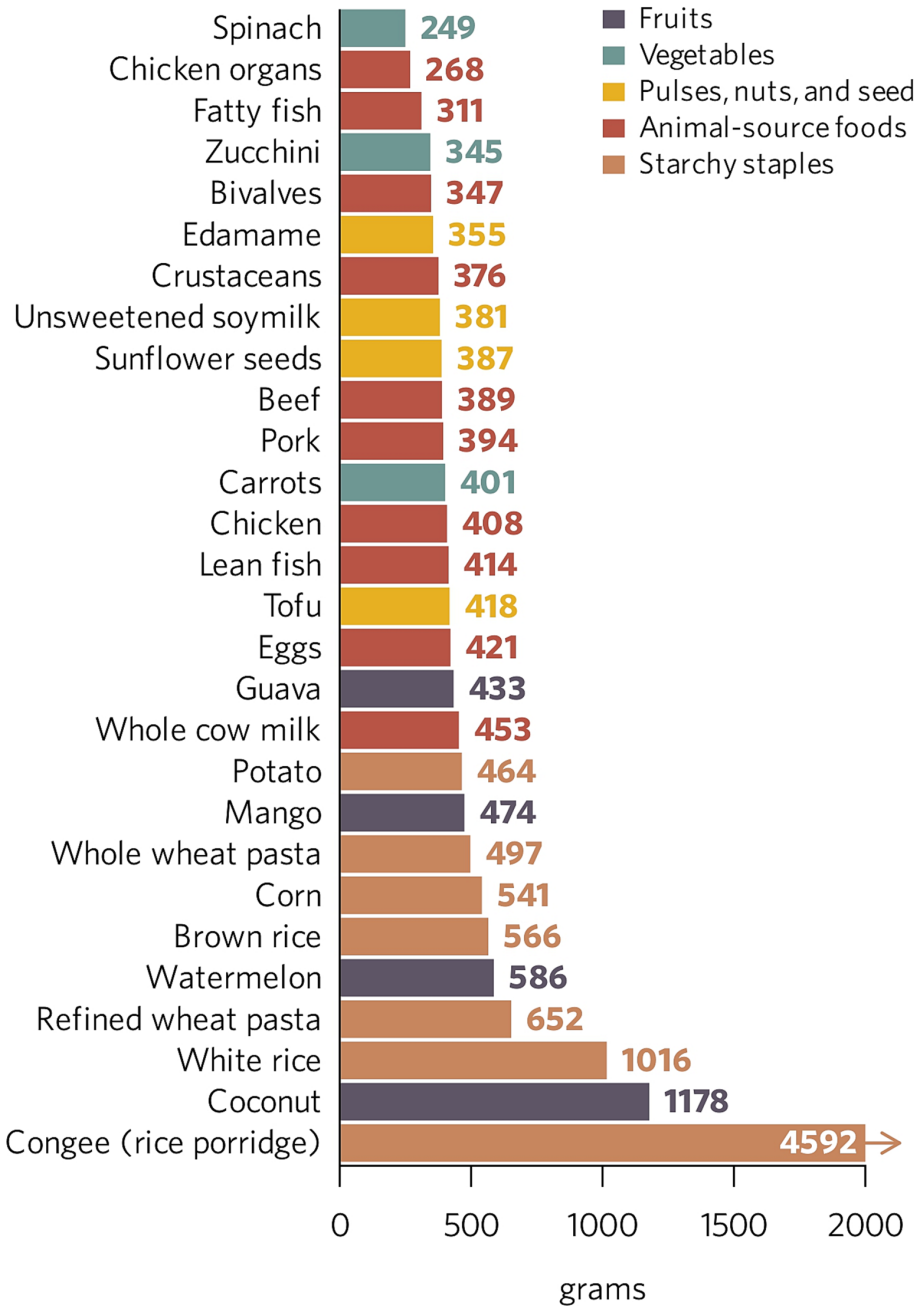
Amount (grams) of common Indonesian foods needed to achieve equivalent Nutritional Value Scores – in this case, an NVS of 100. The NVS rates foods on a scale of 1 to 100, where 1 represents the least nutritious food and 100 the most nutritious one (12). While this ranking facilitates both within- (e.g., different vegetables) and cross-category (e.g., vegetables vs. starchy staples) comparisons of foods’ nutritional value, it does not imply direct interchangeability of items in meals or diets. Foods play distinct roles in a diet, and all recommended food groups consumed in adequate proportions (i.e., as per dietary guidelines) should be part of healthy and sustainable eating patterns. Reproduced from Ortenzi et al (13), licensed under CC BY-NC-SA 4.0 IGO.

The NVS, a novel nutrient profiling system specifically designed for sustainability assessments, incorporates both nutrients of global health priority and dietary factors predictive of noncommunicable disease (NCD) risk, and adjusts for protein digestibility and bioavailability of iron and zinc – making it suitable for use in HIC and LMIC settings alike (12). When used as a functional unit, the NVS enables more nutritionally relevant comparisons of foods’ environmental footprints than traditional mass-, energy-, or single-nutrient-based denominators (e.g., 1 kg, 1,000 Calories, or 100 g of protein). By holistically capturing foods’ nutritional value, the NVS positions itself as a promising index for quantifying foods’ environmental impacts relative to their contribution to global dietary adequacy and quality. For example, white rice and fatty fish have widely different nutrient profiles and play distinct roles in diets (i.e., source of energy from carbohydrates vs. source of protein, omega-3 s, and micronutrients); therefore, directly comparing their environmental footprints on a per-kilogram basis is not nutritionally robust. Even when comparing food items within the *same* category (e.g., two vegetables like spinach and carrots), mass- or energy-based metrics can yield misleading conclusions regarding their *enviro-nutritional* efficiency. For instance, a significantly smaller quantity of spinach (~250 g) achieves an equivalent NVS to a larger portion of carrots (~400 g), highlighting the difference in nutrient density and NCD-protective effects between these foods (Figure 1) (13). Further, taken from the perspective of LCA best practice (ISO, 2006), the *function* of distinct food items (within or across categories) at the point of consumption cannot be captured by mass, and only partially by energy, without accounting for broader nutritional considerations in parallel (18).

Empirical applications of nLCA in Indonesia, Kenya, and Rwanda demonstrated how locally adapted data and context-specific impact category weights can identify ‘best-bet’ foods for delivering high nutritional value at relatively low environmental costs. Our analyses revealed that while plant-source foods often perform well, certain minimally processed, nutrient-dense foods of animal origin – including organ meats, fish and seafood, eggs, and some dairy products – can be competitive when assessed per unit of nutritional value, particularly if consumed in line with national dietary recommendations (13). These findings challenge simplistic dichotomies and point to the need for nuanced, context-sensitive guidance regarding consumption of plant and animal products within healthy and sustainable diets (4–11).

## 3 Current challenges and implications for decision-making

From a methodological perspective, we highlight significant limitations surrounding functional unit selection, assessment scope, approach standardisation, transparency, replicability, results interpretation, and uncertainty communication (4–11).

Unlike other recent adaptations of, and advances in LCA, such as prospective and spatial LCA (19–22), nutritional LCA can introduce health-based narratives which feed into, either directly or indirectly, recommendations for individual- and/or population-level dietary changes (23, 24). The proposed consumption shifts may have significant implications for nutrition and health outcomes. Therefore, our symposium stressed the importance of scientific and clinical due diligence, as well as local health professional engagement, and called for caution against overinterpreting small differences in *enviro-nutritional* footprint scores given the inherent uncertainties in (n)LCA modelling (25).

**Figure 2:**
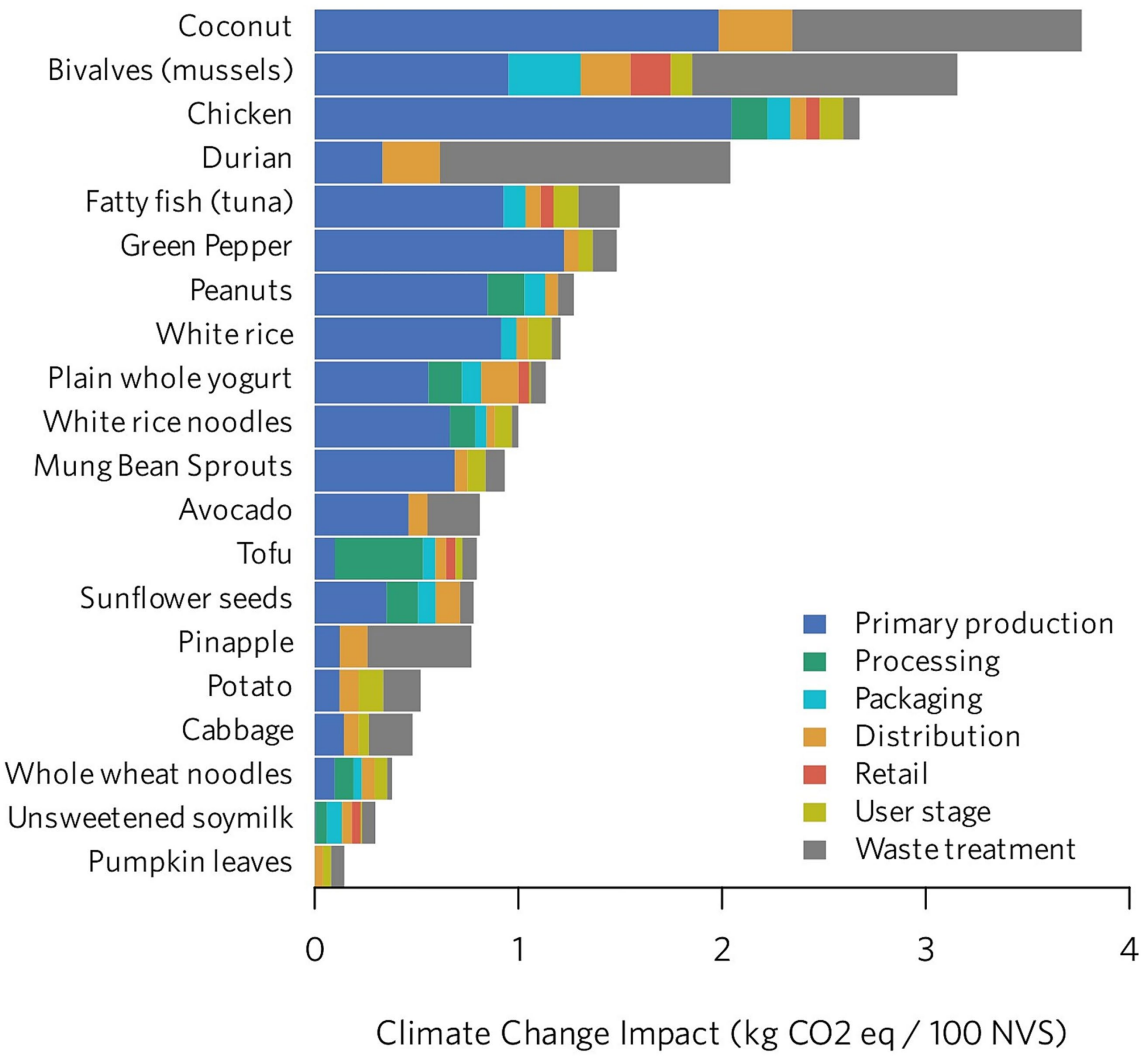
Contribution of different lifecycle stages to climate change impacts (expressed as kg CO_2_ eq/100 NVS) for a selection of foods commonly consumed in Indonesia. The NVS (specifically, an NVS of 100) was used as the nutritional functional unit for this analysis (12). Reproduced from Ortenzi et al (13), licensed under CC BY-NC-SA 4.0 IGO.

The utility of nLCA-derived insights extends across policy, programmatic, and industrial spheres, from informing sustainable dietary guidelines and optimised public procurement strategies, to competitive product benchmarking, consumer-demand generation initiatives, and the identification of environmental ‘hotspots’ throughout supply chains (Figure 2) (13). For example, recent work led by the Global Alliance for Improved Nutrition in Indonesia illustrated how nLCA findings have already been leveraged to influence (sub)national food systems policy and action planning, and to incentivise innovation in local food production (13, 26, 27). When it comes to informing public and corporate decision-making, clear results communication is essential: while aggregated *enviro-nutritional* impact scores can aid interpretation by diverse (technical and non-) stakeholders, they risk obscuring the relative importance of and potential trade-offs among individual environmental categories. This concern is of notable relevance for spatially-dependent impact categories (e.g., water and soil pollution potentials, including eutrophication and acidification) (4, 13).

In addition to these conceptual and interpretative issues, there are persistent, wide-ranging data gaps, particularly regarding the availability and geographic representativeness of both nutritional and environmental datasets, which are unevenly distributed across regions, value chains, and production systems (4–11).

Current evidence emphasises that transdisciplinary and multisectoral collaboration is essential to improve data quality and spatial granularity, as well as to harmonise methods and ensure appropriate use of results, toward enhancing local relevance of analytical outputs, enabling cross-study comparisons, and facilitating coherent policy and intervention design in food systems (4–11).

## 4 Discussion and future directions in enviro-nutritional modelling

The open panel discussion that followed the session’s structured presentations reflected the diverse composition of the audience, with contributions from public health professionals, nutrition experts, environmental scientists, policymakers, programme managers, and private sector representatives. Questions centred on the feasibility of applying complex nutrient profiling systems and nLCA models in data-scarce settings; the poorly captured role of cultural acceptability and socio-economic impacts in defining sustainable diets; and the potential for nLCA to guide reformulation and innovation in the food industry.

Reflecting the symposium’s consensus, we suggest that advancing nLCA requires progress in three key areas to unlock its full potential as an evidence-based tool to inform high-stakes food system decision-making. In the *nutritional domain*, methodological refinement must prioritise (i) developing practically viable yet comprehensive nutrient indices that are fit-for-purpose and contextually relevant, and (ii) improving the quality and regional coverage of dietary intake and food composition datasets. Simultaneously, research in the *environmental domain* must also address data infrastructure gaps, as well as expand assessment scopes – e.g., to include a broader range of value chains, production systems, and impact categories – and parameter-level uncertainty measurements. Building on these disciplinary foundations, future *integrated assessments* should focus on (i) harmonising functional units and analytical approaches, and (ii) incorporating other sustainability dimensions (i.e., socio-economic and cultural) within the same LCA framework, to generate consistent, transparent, and holistic metrics (4–11, 17).

In alignment with the broader literature, all invited experts and session participants emphasised that the current nLCA evidence base is heavily skewed toward HICs. Closing the ‘data equity’ gap – particularly across Sub-Saharan Africa and parts of Asia – is essential for ensuring that nLCA results reflect local agroecological conditions, production practices, and food consumption patterns. Failure to improve geographic granularity may lead to ‘one-size-fits-all’ policy decisions in LMICs based on models derived from HIC archetypes (4–11).

Moreover, future nLCA efforts should evolve beyond attributional, climate-centric assessments to embrace prospective and geospatial modelling (19, 20). This transition will allow researchers to estimate the nutrient provision capacity of alternative farming systems relative to the arable land required, offering a more sophisticated understanding of land-use efficiency (21, 22).

Further developments are critically needed to consider broader sustainability aspects – such as biodiversity loss, animal welfare, and the sociocultural and livelihoods implications of supply chains – and to explore meal- and whole diet-level (in addition to product-level) nLCA models, as well as system-wide evaluations where food production and consumption perspectives are integrated. For instance, applying nLCA methodology to pre-defined dietary scenarios (e.g., national average consumption patterns, dietary guidelines, school canteen menus) may offer more comprehensive, policy-relevant insights than single-product assessments, as it inherently accounts for nutritional complementary of food items while measuring environmental and social impacts (4, 28–30). Nevertheless, granular comparisons both within and across food groups remain essential for benchmarking specific commodities and guiding targeted food-based interventions.

The first-time inclusion of a dedicated nLCA symposium at such a prominent nutrition science conference signals significant progress toward mainstreaming environmental footprint evaluations in global food systems research and policy agendas. However, to prevent the fragmentation and incomparability of findings from different studies, there is an urgent need to harmonise nutritional functional units and establish transparent reporting standards. This is especially important given the recently introduced mandatory reporting policies in many countries and regions, such as the European Union’s Corporate Sustainability Reporting Directive and the Australian Sustainability Reporting Standards (31, 32).

## 5 Conclusion

By combining environmental burden and nutritional value quantification within the same analytical frame, nLCA offers a route to more coherent and holistic food systems policies and interventions. The evidence synthesised in this Perspective illustrates that nLCA represents a major methodological advancement from mass- or energy-based assessments, which often penalise nutrient-rich foods with great potential to positively contribute to global dietary adequacy and quality. By shifting to nutrition-based denominators, we can better characterise the synergistic effects of ‘best-bet’ foods that support both human and planetary health.

While methodological limitations remain significant, the momentum established at the IUNS-ICN 2025 session demonstrates a clear appetite for interdisciplinary alignment. The challenge for the coming decade is not merely technical, but also ethical and political: we must leverage *enviro-nutritional* modelling in ways that respect local sociocultural realities and promote economic prosperity while contributing to global sustainability and health targets.

Ultimately, embedding nLCA within the food and nutrition science field is no longer a ‘niche’ research interest; it is a requirement for designing future-proof food systems. For researchers, policymakers, and industry actors alike, nLCA can offer the cross-sectoral language necessary to reconcile the often-conflicting goals of agricultural production, environmental protection, and public health nutrition.

## Data Availability

Publicly available datasets were analyzed in this study. This data can be found here: all data referred to in this perspective can be found in previous publications (see citations) and in the following repository: McAuliffe, G, Ortenzi, F, McLaren, S, Ponsioen, T, Beal, T. Underlying Nutritional Life Cycle Assessment (nLCA) inventory data applicable to an Indonesian case study of the Nutritional Value Score (NVS). Mendeley Data. 2025; V1. Available at: https://data.mendeley.com/datasets/2fzwjty3jc/1.
