# Correction: Nutritional life cycle assessment for healthy and sustainable food systems: evidence and policy insights from Africa and Asia

**DOI:** 10.3389/fnut.2026.1814004

**Published:** 2026-03-04

**Authors:** Graham A. McAuliffe, Flaminia Ortenzi, Jolieke C. van der Pols, Thomas Nemecek, Jessica Colston, Ty Beal

**Affiliations:** 1 Harper Food Innovation, Harper Adams University, Newport, United Kingdom; 2 Knowledge Leadership, Global Alliance for Improved Nutrition (GAIN), Geneva, Switzerland; 3 School of Exercise and Nutrition Sciences and the Centre for Agriculture and the Bioeconomy, Queensland University of Technology (QUT), Brisbane, QLD, Australia; 4 Life Cycle Assessment Research Group, Agroscope, Zurich, Switzerland; 5 Programme Services Team, Global Alliance for Improved Nutrition (GAIN), London, United Kingdom; 6 Knowledge Leadership, Global Alliance for Improved Nutrition (GAIN), Washington, DC, United States

**Keywords:** environmental impact, food policy, low- and middle-income countries, nLCA, nutrient profiling, nutritional life cycle assessment, sustainable food systems

The Acknowledgment statement was incorrect as published. The incorrect statement read as, “We would like to thank the sponsor of the symposium (GAIN), the conference's technical and logistics staff, the Chair and invited experts, and all session participants for their contribution to the success of the event.” The correct statement should read as, “We would like to thank Dr. Kerstin Damerau for leading the nutritional Life Cycle Assessment modeling work in Kenya and Rwanda referred to in this Perspective – related scientific manuscript upcoming. Additionally, we would like to thank the sponsor of the symposium (GAIN), the conference's technical and logistics staff, the Chair and invited experts, and all session participants for their contribution to the success of the event.”

The original version of this article has been updated.

